# Effects of 4G Long-Term Evolution Electromagnetic Fields on Thyroid Hormone Dysfunction and Behavioral Changes in Adolescent Male Mice

**DOI:** 10.3390/ijms252010875

**Published:** 2024-10-10

**Authors:** Hyun-Yong Kim, Yeonghoon Son, Ye Ji Jeong, Soo-Ho Lee, Nam Kim, Young Hwan Ahn, Sang Bong Jeon, Hyung-Do Choi, Hae-June Lee

**Affiliations:** 1 Division of Radiation Biomedical Research, Korea Institute of Radiological & Medical Sciences (KIRAMS), Seoul 01812, Republic of Korea; khy9514@kbiohealth.kr (H.-Y.K.); sonyh@kirams.re.kr (Y.S.); whyj0914@kirams.re.kr (Y.J.J.); skarn88@naver.com (S.-H.L.); 2 New Drug Development Center, Osong Medical Innovation Foundation, Cheongju 28160, Republic of Korea; 3 School of Electrical and Computer Engineering, Chungbuk National University, Cheongju 28466, Republic of Korea; namkim@chungbuk.ac.kr; 4 Department of Neurosurgery, Ajou University School of Medicine, Suwon 16499, Republic of Korea; yhahn@ajou.ac.kr; 5 Department of EMF Research Team, Electronics and Telecommunication Research Institute, Daejon 34129, Republic of Korea; sbjeon@etri.re.kr

**Keywords:** adolescence, HPT axis, LTE signal, mice, RF-EMF exposure, thyroid hormone

## Abstract

Radiofrequency electromagnetic fields (RF-EMFs) can penetrate tissues and potentially influence endocrine and brain development. Despite increased mobile phone use among children and adolescents, the long-term effects of RF-EMF exposure on brain and endocrine development remain unclear. This study investigated the effects of long-term evolution band (LTE) EMF exposure on thyroid hormone levels, crucial for metabolism, growth, and development. Four-week-old male mice (C57BL/6) were exposed to LTE EMF (whole-body average specific absorption rate [SAR] 4 W/kg) or a positive control (lead; Pb, 300 ppm in drinking water) for 4 weeks. Subsequently, the mice underwent behavioral tests including open field, marble burying, and nest building. Blood pituitary and thyroid hormone levels, and thyroid hormone-regulating genes within the hypothalamus–pituitary–thyroid (HPT) axis were analyzed. LTE exposure increased T3 levels, while Pb exposure elevated T3 and T4 and decreased ACTH levels. The LTE EMF group showed no gene expression alterations in the thyroid and pituitary glands, but hypothalamic *Dio2* and *Dio3* expressions were significantly reduced compared to that in the sham-exposed group. Pb exposure altered the hypothalamic mRNA levels of *Oatp1c1* and *Trh*, pituitary mRNA of *Trhr*, and *Tpo* and *Tg* expression in the thyroid. In conclusion, LTE EMF exposure altered hypothalamic *Dio2* and *Dio3* expression, potentially impacting the HPT axis function. Further research is needed to explore RF-EMF’s impacts on the endocrine system.

## 1. Introduction

With the evolution of telecommunication technology, mobile communication devices now use a newer generation of wireless broadband to improve the capacity and speed of data transfer. Mobile phone electromagnetic fields (EMFs) have rapidly advanced from the first generation (1G), which used analog signals for voice calls only, to the fifth generation (5G), which has enhanced data transfer speed and supports quality voice calls and improved internet or streaming service [1]. Currently, 4G LTE (long-term evolution) remains the most widely adopted mobile communication technology, used in over 240 countries. The introduction of 5G aims to address the limitations of 4G, and it is gaining global traction. As communication technology evolves and different frequencies of EMFs are applied, it is crucial to investigate their biological impacts.

The increasing mobile phone use by young users raises concerns about the impact of radiofrequency electromagnetic fields (RF-EMFs) on children and adolescents. These groups are in critical stages of development, and their developing brains, with higher conductivity and a thinner skull than adults, may be more susceptible to RF-EMFs [2]. Epidemiological studies on the impact of RF-EMF on thyroid hormones are limited and their results are contradictory. For example, Mortazav et al. investigated the effect of RF-EMF induced by GSM mobile phones on TSH and thyroid hormones in 77 healthy students (ages 19–29) and found no significant difference in T3 and T4 levels in daily 20 min user, users with more than 120 min exposure, and non-users [3]. However, both mobile phone user groups showed elevated TSH levels compared to the non-users. A cross-sectional study on 83 undergraduate students found that an increase in TSH correlated with SAR values (ranged from 0.67 to 1.19) and the duration of daily mobile phone use (1 to 3 h/day) [4]. Another study, which examined different age groups (14–22 years and 25–60 years) exposed to mobile phones or base stations for 1 to 6 years, found significant decreases in T3 and T4 levels [5] in both age groups. While previous research suggests that RF-EMF exposure may induce imbalance or disruption in thyroid hormone levels, the current epidemiological and experimental human studies on cognition, neurodevelopment, endocrine system, and behaviors in children and adolescents exposed to RF-EMFs from wireless communication devices have not reached a definitive conclusion about the risk posed by RF-EMFs [6,7,8]. Challenges such as a limited number of studies, inconsistent data, ethical issues, and a lack of well-controlled EMF experiments contribute to this uncertainty. Thus, experimental data from animal studies, especially those modeling children and adolescents, are needed, even if the results from these animal studies may not perfectly match human outcomes.

Mobile phones are often used in positions close to the head (brain) and thyroid glands. The thyroid hormones (T3 and T4) regulated by the hypothalamus–pituitary–thyroid (HPT) axis are essential for metabolic regulation, growth, and development [9,10]. Experimental studies on the effect of RF-EMFs on thyroid glands and function in adult animals have yielded conflicting results, with some studies reporting no effects [11,12] and others showing significant alteration in T3 and T4 levels [13,14,15]. However, there is a lack of research on the impact of RF-EMFs on thyroid hormones in young and adolescent animals.

In this study, we focused on LTE EMFs and their impact on thyroid hormones and thyroid hormone-regulating factors during childhood and adolescence. We assessed the effects on thyroid hormone levels, thyroid gland histopathology, and gene expression involved in thyroid hormone regulation within the HPT axis in adolescent male mice. Using male C57BL/6 mice exposed to 1760 MHz frequency LTE EMFs (with a whole-body average specific absorption rate (SAR) of 4 W/kg) and lead (Pb, 300 ppm in drinking water), the study aimed to delineate the specific effects attributable to RF-EMFs. Lead was selected owing to its established influence on thyroid hormones, providing a positive control.

## 2. Results

### 2.1. Body Weight Change

**Figure 1:**
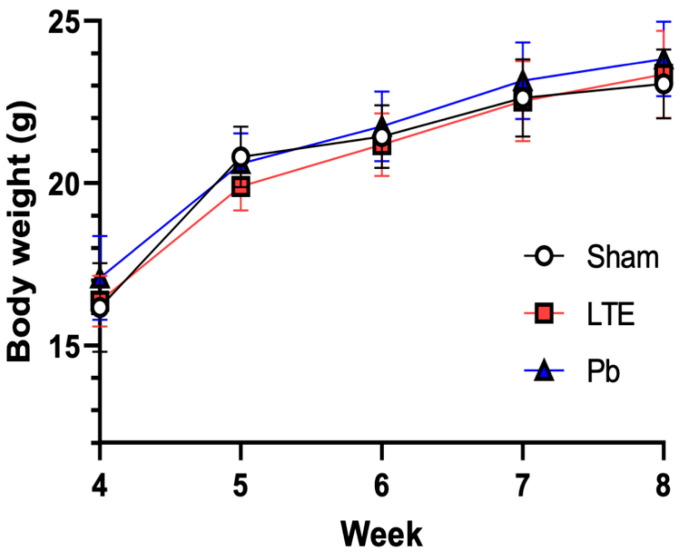
Measurement of body weight. All animals subjected to LTE-RF-EMF (LTE) or Pb exposure were weighed weekly for 4 weeks.

Body weight was measured in each group of mice over 4 weeks under LTE- and Pb-exposure conditions. No significant changes in body weight were observed in either the LTE or Pb exposure groups compared to the sham group (Figure 1). To evaluate statistical significance, we analyzed the weight gain data using a multivariate analysis of variance (MANOVA), with treatment (LTE or Pb exposure) as the between-subject factor and time as the within-subject factor. The analysis showed no significant effect of treatment (F(2,27) = 6.29, *p* = 0.2638) or any interaction between the treatment and time (F(8108) = 1.23, *p* = 0.2899). However, there was a significant effect of time on body weight (F(4108) = 376, *p* < 0.0001), indicating natural weight progression over time independent of treatment conditions. Additionally, the monitoring of body temperature showed no significant changes in temperature during LTE exposure; the temperature was 36.58 ± 0.56 °C before exposure and 36.28 ± 0.55 °C after exposure.

### 2.2. Effect of LTE or Pb Exposure on Behavior

**Figure 2:**
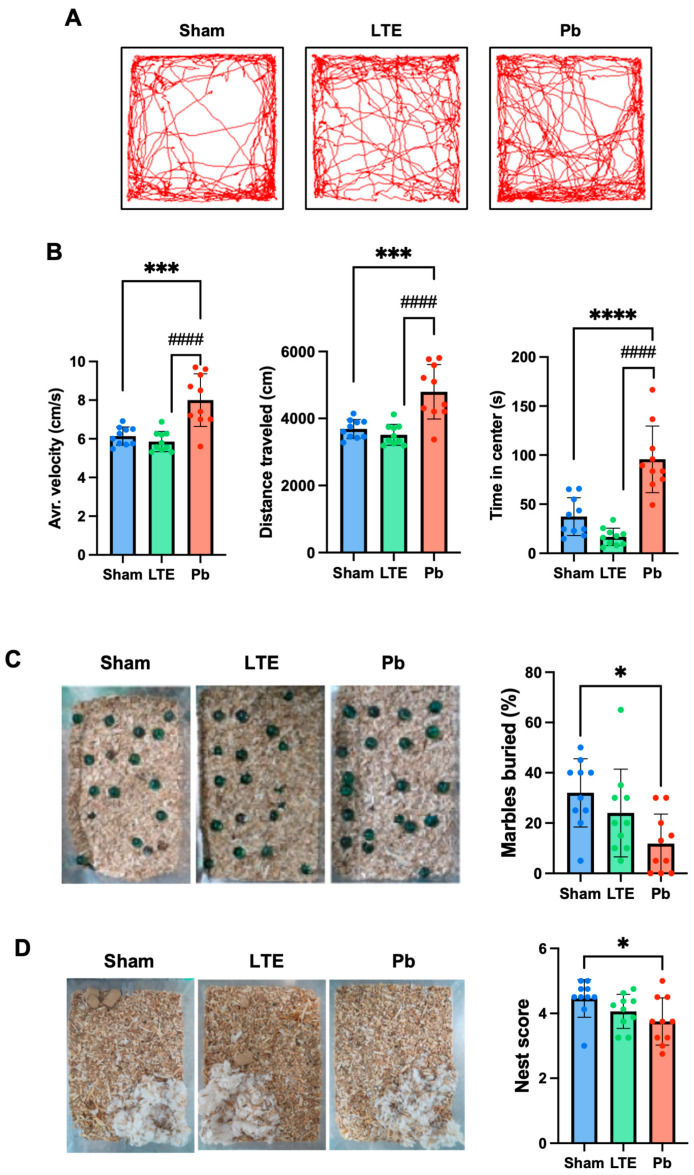
Evaluation of behavioral changes in the mice exposed to LTE or Pb treatments during adolescence. (**A**) The representative traces of movement measured in the open-field test. (**B**) Measured average velocity, distance traveled, and activity and time in the central zone. (**C**) The results of the marble-burying test with representative photos and quantification in the mice exposed to sham, LTE, or Pb treatments during adolescence. (**D**) The representative photos and scores of the built nests by the mice exposed to sham, LTE, or Pb treatments during adolescence. The data are presented as mean ± standard deviation (n = 10). * *p* < 0.05, *** *p* < 0.001, and **** *p* < 0.0001 versus sham; ^####^ *p* < 0.0001 versus LTE exposure.

In the open-field test, the LTE exposure group showed a decrease in velocity and distance traveled, but these differences were not statistically significant compared to the sham group. LTE exposure during adolescence did not significantly alter locomotor activity, anxiety-like behaviors or mood, and general health represented by the open-field, marble-burying, and nest-building tests (Figure 2). However, Pb exposure led to behavioral disorders such as hyperactivity, indicated by increased velocity (F(2,27) = 17.39, *p* = 0.0002 vs. sham, *p* < 0.0001 vs. LTE) and total distance traveled (F(2,27) = 17.38, *p* = 0.0002 vs. sham, *p* < 0.0001 vs. LTE) in the open-field test. Pb exposure also decreased anxiety behaviors (more time spent in the central area of the open field, F(2,27) = 31.44, *p* < 0.0001 vs. sham, *p* < 0.0001 vs. LTE), reduced the number of buried marbles in the marble-burying test (F(2,27) = 4.944, *p* = 0.0115), and resulted in incomplete nests in the nest-building test (F(2,27) = 3.367, *p* = 0.0394) in comparison to the performance of the sham group. These results indicated that while the LTE exposure did not induce behavioral dysfunction, the Pb exposure significantly influenced the behaviors of the adolescent mice.

### 2.3. Effect of LTE and Pb Exposure on Thyroid and Other Hormones during Adolescence

**Figure 3:**
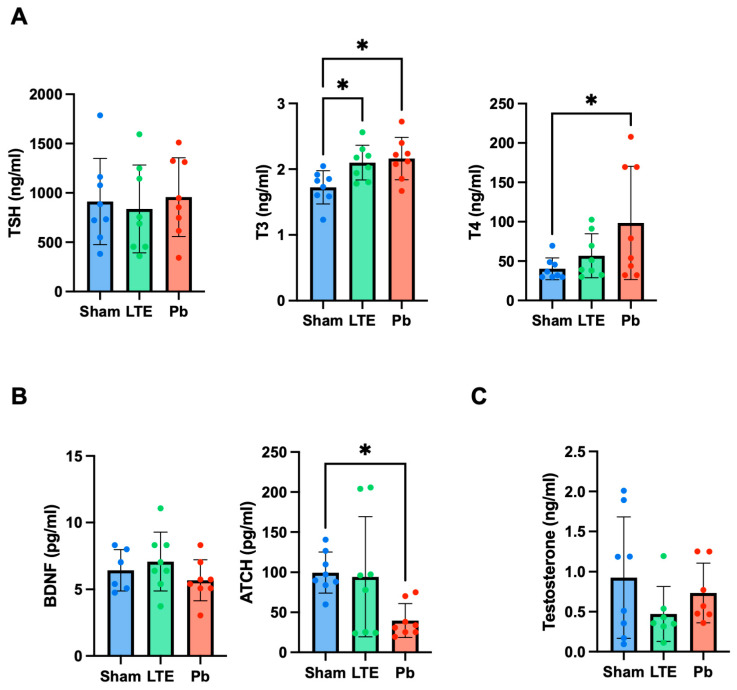
Analysis of the circulating hormone levels following LTE or Pb exposure during adolescence in the C57BL/6 mice. (**A**) Thyroid hormones: TSH, T3, and T4. (**B**) Pituitary hormones: brain-derived neurotrophic factor (BDNF) and adrenocorticotropic hormone (ACTH). (**C**) The testosterone levels. The data are presented as mean ± standard deviation (n = 7–8). * *p* < 0.05 versus sham.

The LTE exposure during adolescence showed no changes in the thyroid-stimulating hormone (TSH) and T4 levels, while the T3 levels were significantly increased (F(2,21) = 5.679, *p* = 0.0364) compared to the sham-exposed group (Figure 3). Pb exposure did not affect the TSH levels; however, both the T3 and T4 levels were significantly increased compared to those of the sham group (F(2,21) = 5.679, *p* = 0.0139 for T3, F(2,21 = 3.523), *p* = 0.0446 for T4) (Figure 3A).

As the LTE exposure increased T3 hormone levels, we further investigated the other hormones. Testosterone levels, a male reproductive hormone, showed no alterations following either the LTE or Pb exposure (Figure 3B). Subsequently, other pituitary hormones BDNF and ACTH were analyzed. The LTE exposure did not influence either BDNF or ACTH levels, whereas the Pb exposure led to decreases in ACTH levels compared to those of the sham group (F(2,21) = 3.917, *p* = 0.0495). These suggested that LTE EMF may influence thyroid hormone levels, particularly T3, while Pb exposure during adolescence can have detrimental effects on the HPT axis, leading to an imbalance of thyroid hormones. 

### 2.4. Effect of LTE and Pb Exposure on Gene Expression Related to Thyroid Metabolism

**Figure 4:**
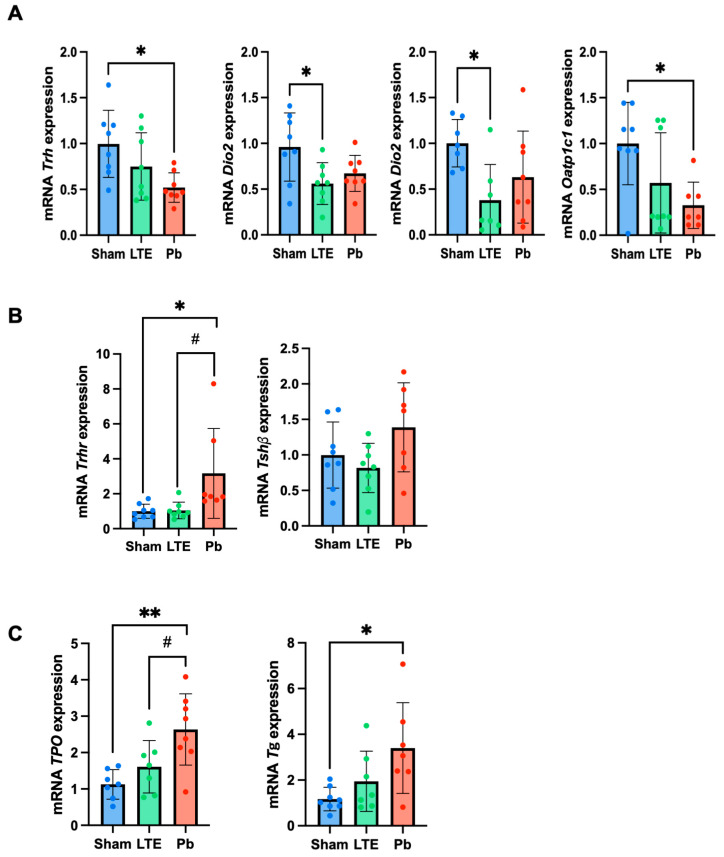
RT-qPCR analysis of the thyroid hormone-regulating genes in the hypothalamic–pituitary–thyroid (HPT) axis. The relative mRNA expression levels of *Trh, Dio2, Dio3*, and *Oatp1c1* in the hypothalamus (**A**); *Trhr* and *Tshβ* in the pituitary gland (**B**); and *Tpo* and *Tg* in the thyroid glands (**C**). The data are presented as mean ± standard deviation (n = 7–8). * *p* < 0.05 and ** *p* < 0.01 versus sham; ^#^ *p* < 0.05 versus LTE exposure.

Given the increases in T3 and/or T4 hormones due to the Pb or LTE exposure, we next evaluated the thyroid hormone-regulating genes in the hypothalamus, pituitary, and thyroid glands—collectively known as the HPT axis—using qRT-PCR. The LTE exposure reduced the mRNA expression of *Dio2* (56 ± 22%, F(2,21) = 4.418, *p* = 0.0233 vs. sham) and *Dio3* (34 ± 41%, F(2,19) = 4.232, *p* = 0.0243 vs. sham) in the hypothalamus, but did not affect the mRNA levels of *Trh* and *Oatp1c1* (Figure 4A). In the pituitary gland, *Trhr* and *Tshβ* mRNA levels remained unchanged by the LTE exposure (Figure 4B). While LTE did not alter *Tg* and *Tpo* mRNA expression in the thyroid glands, increasing trends were observed, though not statistically significant due to high variability in each group (Figure 4C). However, the Pb exposure significantly reduced the mRNA levels of *Trh* (52 ± 16%, F(2,21) = 4.617, *p* = 0.0165 vs. sham) and *Oatp1c1* (33 ± 25%, F(2,20) = 4.524, *p* = 0.0208 vs. sham) in the hypothalamus (Figure 4A). In contrast, the *Trhr* gene expression levels in the pituitary gland were elevated (3.1-fold, F(2,20) = 5.280, *p* = 0.0243 vs. sham, *p* = 0.0277 vs. LTE). Additionally, the mRNA expression of *Tpo* (2.6-fold, F(2,19) = 7.898, *p* = 0.0029 vs. sham, *p* = 0.0423 vs. LTE) and *Tg* (3.7-fold, F(2,19) = 5.006, *p* = 0.0144 vs. sham) notably increased in the thyroid glands of the Pb-exposed mice compared to those of the sham group. These results indicate that Pb exposure during adolescence significantly disrupts the HPT axis, while LTE exposure affects the T3- and T4-converting enzymes Dio2 and Dio3 in the hypothalamus, suggesting that LTE could also affect thyroid hormonal regulation.

## 3. Discussion

The close proximity of mobile phone use to the ear, a location near the brainstem, including the hypothalamus, raises significant concerns about the potential effects of RF-EMF exposure on the endocrine system. This is especially important for children and adolescents, who are frequent users of mobile devices and are more susceptible to RF-EMF exposure. The thyroid gland, located superficially in the neck, could be particularly vulnerable to RF-EMFs emitted from mobile devices. Thyroid hormones are crucial for normal brain development, cognitive function, and emotional regulation [16], and their secretion is regulated by the HPT axis. In this study, we elucidated the effects of RF-EMF exposure on thyroid hormone levels and the HPT axis in adolescent mice, exploring possible health risks.

In the present study, to clarify the impact of RF-EMF exposure during adolescence, we started LTE RF-EMF exposure in young mice at 4 weeks of age, as adolescence in mice is generally considered to be between weaning (3–4 weeks old) and young adulthood (9–10 weeks) [17,18]. The mice were exposed to LTE signals at an SAR level of 4 W/kg, with whole-body average exposure for 8 h daily over 4 weeks. Our results showed that T3 levels were significantly elevated in the mice exposed to LTE during adolescence compared to the sham group, while TSH and T4 levels remained unchanged. T3 is the active form of thyroid hormones and plays a critical role in growth and neurodevelopment. Thus, we assessed body weights and behavioral changes in each group. However, the male mice did not show any significant difference in body weight or abnormal behaviors in the open-field, marble-buying, and nest-building tests. A previous study by Sinha [15] exposed male rats, aged 4–5 weeks, to 2.45 GHz RF-EMF for 16 or 21 days (2 h/day), with a whole-body average SAR of 0.036 W/kg. They found decreased T3 levels at 16 and 21 days, increased T4 levels at 21 days, and no changes in TSH levels. Additionally, they observed hyperactive aggressive behaviors in open-field tests and the elevated plus maze. In contrast, Kim et al. [11] exposed 6-week-old rats to 915 MHz RFID signals (8 h/d, 5 d/week, for 2–16 weeks at a whole-body average (WBA) SAR of 4 W/kg) and found no effects on T3 and TSH over time, but a decline in T4 at 4 weeks of exposure, with no morphological changes in the thyroid. While thyroid hormone-related diseases such as hypothyroidism or hyperthyroidism are associated with thyroid dysfunction, there was inconsistency in the effects of RF-EMF exposure on thyroid hormone levels in animal studies, including our present study. This inconsistency may be due to the relatively low biological impact of RF-EMFs compared to the other types of electromagnetic fields, such as extremely low-frequency or ionizing radiation, which may not be sufficient to induce irreversible changes in tissues or organs. Alternatively, the slight increase in T3 hormone levels observed in response to the LTE EMF exposure may not be enough to cause behavioral disorders in animals. Additionally, we investigated hormonal systems beyond the HPT axis due to the critical developmental period during which the mice were exposed to RF-EMF (ages 4 to 8 weeks). During adolescence, multiple hormonal systems, including the HPT axis, are essential for development. To assess the broader health impacts of RF-EMF on adolescent animals, we selected BDNF (brain), ACTH (pituitary), and testosterone (peripheral organ) as key indicators, representing important physiological functions during adolescence. RF-EMF showed no alteration in these indicators.

However, our findings suggest that LTE EMFs during adolescence can lead to an imbalance in thyroid hormone homeostasis, specifically by altering T3 hormone levels. To further elucidate the impact of LTE EMF on T3 levels, we conducted an additional analysis of the hypothalamic–pituitary–thyroid (HPT) axis. The HPT axis is a critical endocrine pathway involving the hypothalamus, pituitary gland, and thyroid gland [9] which regulates thyroid hormone production and release. The hypothalamus secretes thyrotropin-releasing hormone (TRH), which prompts the pituitary to release TSH, in turn stimulating the thyroid gland to produce T3 and T4. T3 and T4 regulate hypothalamic TRH and pituitary TSH via negative feedback. T4 is primarily transported through Oatp1c1 to the brain, where it is catalyzed to T3 by the Dio2. Tissue T3 is transported through monocarboxylic acid transporter 8 (Mct8) and metabolized to T2 by Dio3. In our study, the LTE exposure resulted in elevated T3 levels and the reduced expression of *Dio2* and *Dio3*. The decrease in *Dio2* gene expression (*p* < 0.05) may be due to negative feedback from increased T3 levels in the tissue. The LTE exposure also led to the downregulation of mRNA Dio3 expression, potentially maintaining high T3 levels. LTE did not alter the other HPT-regulating genes, including *Oatp1c1*, *Tg*, and *Tpo*. Our findings suggest that RF-EMF exposure affects the HPT axis at the molecular levels. This finding concurs with a previous study Zufry et al., [13] which also suggested that RF-EMF exposure (120–150 min daily for 12 weeks) significantly reduced MCT8 protein concentration in the brain and resulted in reduced TSH and T4 levels in serum.

As a well-known neurotoxin, Pb exposure during adolescence led to more pronounced disruptions, including significant increases in T3 and T4 levels [19,20], and notable changes in gene expression related to thyroid metabolism across the HPT axis [21,22]. In our study, the Pb exposure significantly decreased *Trh* and *Oatp1c1* mRNA levels in the hypothalamus, upregulated *Trhr* (thyrotropin-releasing hormone receptor) in the pituitary gland, and increased *TPO* and *Tg* expression in the thyroid gland. These results suggest that LTE EMFs and Pb may influence thyroid hormone levels through distinct mechanisms within the hypothalamic–pituitary–thyroid (HPT) axis. Notably, unlike the LTE EMF exposure, the Pb exposure was associated with a reduction in ACTH levels, implying that Pb might affect additional endocrine pathways during adolescence. Consequently, Pb could disrupt the HPT axis via multiple mechanisms, potentially leading to behavioral alterations, such as increased hyperactivity and decreased anxiety-like behaviors, which were not observed in the animals exposed to LTE EMFs.

The study aimed to assess the potential health effects of LTE EMFs on the endocrine system, particularly focusing on the HPT axis, at exposure levels higher than the ICNIRP limits for humans. According to ICNIRP guidelines [23], a 1 °C rise in body core temperature is considered the threshold for harmful biological effects, which requires an SAR exposure level not exceeding 4 W/kg whole-body SAR in humans. To ensure safety, ICNIRP applies a reduction factor of 10 for occupational exposure, setting the SAR limit at 0.4 W/kg, and a factor of 50 for the general public, setting the limit at 0.08 W/kg. Since the effects of RF-EMF on the endocrine system, particularly the HPT axis, are still unclear, our study employed a 4 W/kg SAR standard to explore potential effects. As preliminary findings indicated some influence of LTE EMFs on the HPT axis, further studies at lower exposure levels below 4 W/kg are necessary.

While our study elucidated the effects of RF-EMF exposure on thyroid hormone levels and the HPT axis in adolescent mice, it is important to note that comprehensive studies investigating RF-EMF’s impact on the HPT axis in children and adolescents remain limited, with only a few studies reporting variations in thyroid hormone levels. Some experimental studies have examined the influence of electromagnetic fields or cell phone usage on thyroid hormone levels in these age groups. For example, one study involved healthy school-age children (12.5 ± 1.5 years) divided into two groups: occasional and regular cell phone users. After a mental stress test and 5 min mobile phone use, thyroid hormones (TSH, T3, and T4), glucose, insulin, and cortisol levels were measured 20 min post-exposure [24]. There were no significant differences in the hormonal and biochemical levels between the two groups. Another study on healthy teenagers (17.4 ± 0.24 years) used electrophotonic imaging to assess the impact of RF-EMF exposure (2100 MHZ, 0.4 W/kg of head SAR and 0.54 W/kg of body SAR) on thyroid gland energy levels [25]. After 15 min of RF-EMF exposure from a mobile phone held 0.5 cm from the ear, the participants showed significantly reduced thyroid gland energy levels compared to the sham group. Although these studies are a useful insight into the effects of RF-EMF on children and adolescents, it is still difficult to draw conclusions due to the limited number of studies and variations in exposure conditions and endpoints. Therefore, prospective studies, particularly those involving children and adolescents, are essential to analyze the long-term effects of RF-EMF exposure on thyroid hormones and the endocrine system.

Our results suggest that RF-EMF exposure during adolescence may impact thyroid hormones and HPT axis function in male mice. However, it also has several limitations. We applied very high SAR compared to typical human exposure scenarios and different exposure levels or patterns might yield different results. And, we accessed thyroid function in young adult mice after 4 weeks of RF-EMF exposure during adolescence. However, any detrimental effects related to RF-EMF exposure may not manifest immediately at 8–9 weeks of age, and behavioral and developmental differences might emerge later. This highlights the need for further studies to investigate potential long-term effects. Additionally, we did not clarify the direct molecular alterations in the HPT axis that lead to changes in thyroid hormones. Therefore, future studies should investigate the underlying mechanisms of RF-EMF-induced changes in the HPT axis, particularly focusing on the role of *Dio2* and *Dio3* in thyroid hormone regulation within the brain. Comprehensive neurobiological assessment and the potential synergic effects with other neuro-environmental risk factors are also necessary to understand the impact of RF-EMFs on children or adolescents.

In summary, this study investigated the impact of RF-EMF on thyroid hormones and the HPT axis in adolescent male mice. While LTE RF-EMF exposure did not induce significant abnormalities in blood T4 levels, TSH levels, or behaviors, it did alter T3 levels in the blood and *Dio2* and *Dio3* gene expression in the hypothalamus, suggesting potential HPT axis disruptions at the molecular level. Pb exposure, serving as a positive control, demonstrated a significant dysregulation of the HPT axis and associated behavioral changes, emphasizing the vulnerability of the developing endocrine system to environmental toxins. These findings highlight the need for further research to comprehensively understand the effects of RF-EMFs on the endocrine system, particularly focusing on vulnerable children and adolescents.

## 4. Materials and Methods

### 4.1. Animals

**Figure 5:**
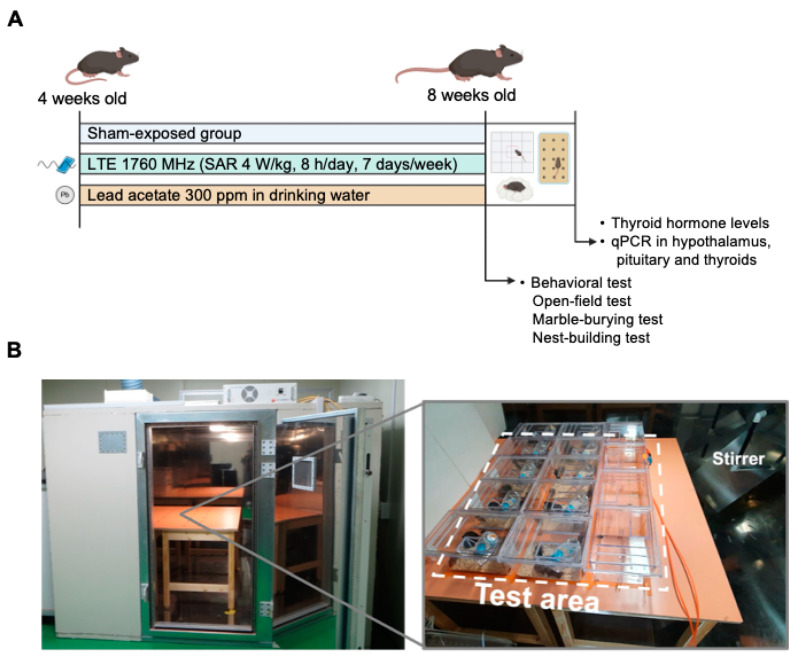
Experimental scheme and in vivo LTE signal exposing chamber. (**A**) LTE and Pb exposure and experimental procedure. (**B**) Image of the reverberation chamber and the positioned cages in the chamber.

Male C57BL/6 mice (4 weeks old) were purchased from Doo Yeol Biotech (Seoul, Republic of Korea). After 1 week acclimatization period, they were randomly assigned to three experimental groups (n = 10): (1) sham group (sham), (2) LTE-RF-EMF exposure group (LTE), and (3) Pb-treated group (Pb) (Figure 5). Pb was administered at a concentration of 300 ppm through drinking water for 4 weeks. All the mice were housed in an animal facility with controlled environmental conditions, including a temperature of 22 ± 2 °C and humidity of 50 ± 10%. They were kept on a 12 h light/dark cycle and provided free access to a standard diet and autoclaved drinking water throughout the study. The body weight of each animal was measured once a week. All the animal experiments were performed in accordance with the guidelines specified by the Institutional Animal Care and Use Committee of the Korea Institute of Radiological and Medical Sciences (KIRAMS) (IACUC permit number: KIRAMS2021-0094 and KIRAMS 2022-0076).

### 4.2. LTE-RF-EMF Exposure System

The animal exposure experiments of LTE EMF signals were conducted in a reverberation chamber (2295 × 2293 × 1470 mm^3^) with a single-mode stirrer. During the exposure, the mice were housed in the same cages used for their normal housing and the lid was replaced by an acryl cover to prevent the scattering of LTE-EMFs (see Figure 5B). Food and water were provided ad libitum. The LTE source signal had a center frequency of 1.76 GHz, a bandwidth of 20 MHz, and a Quadrature Phase shift Keying (QPSK) modulation signal, which was amplified by a high-power amplifier (PCS60WHPA_CW, Kortcom, Anyang, Gyeonggi-do, Republic of Korea) and radiated into the chamber through an antenna inside the reverberation chamber. The output of the high-power amplifier, i.e., the input power, was monitored in real time using a 20 dB directional coupler (778D, Keysight) and a power meter (N1912A, Keysight, Santa Rosa, CA, USA). The electric field uniformity of the reverberation chamber was measured at 24 points inside the working volume. An isotropic field probe (HI-6005; ETS-Lindgren, Cedar Park, TX, USA) was used for electric field measurements. The uniformity was averaged over 1 min and evaluated according to a previous report [26]. The field distribution was well within ± 2 dB inside the working volume. The SAR simulations were conducted using the mouse anatomical model (IT’IS Foundations; Male-OF1-Mouse model). The reverberation chamber was placed in the animal facility, and the temperature and humidity were controlled. The animals were exposed to 1.76 GHz LTE signals according to the following conditions: whole-body average SAR 4 W/kg, 8 h/day, for 4 weeks. The mice of the sham exposure group were placed inside the chambers without LTE signals for equivalent amounts of time. During the RF exposure, the air temperature inside the test area was maintained at 20 ± 3 °C, and the mice were able to move freely. To monitor the thermal effect, the animals’ rectal temperatures (°C) were measured 5 min before the start of the LTE-EMF exposure and immediately after its conclusion using a small-animal thermometer (Test 925; Testo, Lenzkirch, Germany). To minimize stress, two mice from each group were randomly selected once a week for measurement, and all the procedures were conducted by an experienced researcher.

### 4.3. Behavioral Tests

Behavioral tests were conducted during the light phase of the day, specifically between 9:00 a.m. and 12:00 p.m., to minimize variability due to circadian rhythms. Additionally, the order of testing was randomized across the treatment groups to prevent any bias associated with test order or timing.

The open-field test was performed to assess locomotor activity. After the animals were habituated for 30 min in the behavior testing room, each mouse was placed in the center of an acrylic box (45 × 45 × 30 cm) and allowed to explore freely for 10 min. All the movements of each animal were tracked and recorded using a video-tracking system (Viewer3, Biobserve GmbH, Bonn, Germany). The average velocity, total distance traveled, percentage of activity, and time spent in the central zone were measured. The box was cleaned with 70% ethanol after each animal was tested to remove any odor effects.

The marble-burying tests were used to evaluate anxiety-like behaviors and performed as described in a previous study [27] with modifications. Briefly, 15 marbles were given on the surface of a cage filled with woodchip bedding. The mice were allowed to move freely in the cage for 30 min and then the number of marbles that were buried at least two-thirds of the way under woodchip bedding was counted.

The nest-building tests were performed to assess the mood, general health, and welfare of the animals. Each mouse was placed in a cage that contained fresh bedding. The nestlets were placed in each cage for at least 1 h before the dark cycle. The next day, each nest was photographed and scored. Scoring was performed using a previously established grading system [28]. Two independent investigators who were blinded to the animal genotypes performed scoring, and average scores from both investigators were used for statistical analysis.

### 4.4. Determination of Hormone Levels in the Blood

The mice were sacrificed to collect plasma samples by carbon dioxide asphyxiation between 9:00 a.m. and 12:00 p.m. The plasma samples were extracted by centrifuging the blood (3200 rpm for 20 min at 4 °C) and stored at −80 °C for further analysis. T3, T4, and testosterone were analyzed in the mouse plasma samples using the MILLIPLEX Hormone Magnetic Bead Panel (MPTMAH-49K; Millipore, Billerica, MA, USA) and TSH, adrenocorticotropic hormone (ACTH), and brain-derived neurotrophic factor (BDNF) were analyzed using the MILLIPLEX Mouse Pituitary Magnetic Bead Panel (MSHMAG-21K, Millipore). In all the cases, the absorbance of the test samples was measured using a Luminex 200 system (Luminex, Austin, TX, USA). These analyses were performed by KOMA Biotech Inc. (Seoul, Republic of Korea). The data were analyzed using the Xponent 4.2 software (Luminex) by KOMA Biotech Inc (Seoul, Republic of Korea).

### 4.5. Quantitative Real-Time PCR (qRT-PCR)

After euthanizing the animals, the brain tissues were carefully dissected to isolate the hypothalamus and pituitary regions. The thyroid gland was also removed for analysis. All the tissues were immediately frozen and stored at −80 °C until further processing. The total RNA was extracted from these tissues using QIAzol Lysis Reagent (RNeasy lipid tissue mini kit; QIAGEN, Hilden, Germany) following the manufacturer’s protocol, and the concentration of the RNA samples was quantified using a NanoDrop ONE spectrophotometer (Thermo Fisher Scientific, Waltham, MA, USA). The cDNA synthesis was performed using amfiRivert cDNA synthesis platinum master mix (GenDEPOT, Katy, TX, USA). The qRT-PCR was performed using a qPCR Green2X Master Mix kit (m.biotech, Gyeonggi-do, Korea) following the manufacturer’s instructions. The target gene expression was normalized relative to the expression of glyceraldehyde 3-phosphate dehydrogenase (*GAPDH*). The sequences of the forward and reverse primers for the HPT axis-regulating genes are provided in Table 1.

### 4.6. Statistical Analysis

All the data have been presented as mean ± standard deviation (SD). For the weight gain data, a multivariate analysis of variance (MANOVA) for repeated measures was conducted, with one between-subject factor (treatment) and one within-subject factor (time). Additionally, a one-way analysis of variance (ANOVA) was applied to analyze the behavioral, hormonal, and neurochemical data, as appropriate. Post hoc tests were performed where necessary to identify significant group differences. Statistical analyses were performed using GraphPad Prism 9 (GraphPad software, Inc., San Diego, CA, USA). A *p*-value less than 0.05 was considered statistically significant.

## Figures and Tables

**Table 1:** Primers used in qRT-PCR.

Gene Symbols	RefSeq	Sequence
*Trh*	NM_009426	3′-TGTGACTCCTGACCTTCCA-5′
		5′-GGATGCTGGCGTTTTGTG-3′
*Dio2*	NM_010050	3′-CTTCCTCCTAGATGCCTACAAAC-5′
		5′-CTCCGAGGCATAATTGTTACCT-3′
*Dio3*	NM_172119	3′-CACGTGCAAATGCTCCAAAG-5′
		5′-CTCAAGTTAGCCAGACTCAGC-3′
*Oatp1c1*	NM_021471	3′-CCAATGTTACTCCCAGCATCT-5′
		5′-CCAGGAAGACATAAACCCACA-3′
*Trhr*	NM_013696	3′-TGACTCAATCCATCAGAACAAGA-5′
		5′-GGCAAACAGAATTACAACCACT-3′
*Tshβ*	NM_009432	3′-GTCATCACAGCAGTAACTCACT-5′
		5′-CACTCTCTCCTATCCACGTACA-3′
*Tpo*	NM_009417	3′-GTCCTCTGTTTGCATGTATCATTG-5′
		5′-CTTTTCTAGTTCCTGCCTCTGA-3′
*Tg*	NM_009375	3′-TCTCCTGTGATAGTCAAGTCCA-5′
		5′-CACATGAAACCTCTGACTCCA-3′

## Data Availability

The original contributions presented in the study are included in the article, further inquiries can be directed to the corresponding authors.
